# Supplementary material for: Mild Traumatic Brain Injury Induces Transient, Sequential Increases in Proliferation, Neuroblasts/Immature Neurons, and Cell Survival: A Time Course Study in the Male Mouse Dentate Gyrus
Source: Front Neurosci. 2021 Jan 7;14:612749. doi: 10.3389/fnins.2020.612749 (PMC7817782; doi:10.3389/fnins.2020.612749)
Supplement: Supplementary file 1 [file Data_Sheet_1.pdf]

Supplementary Material – Clark et al.

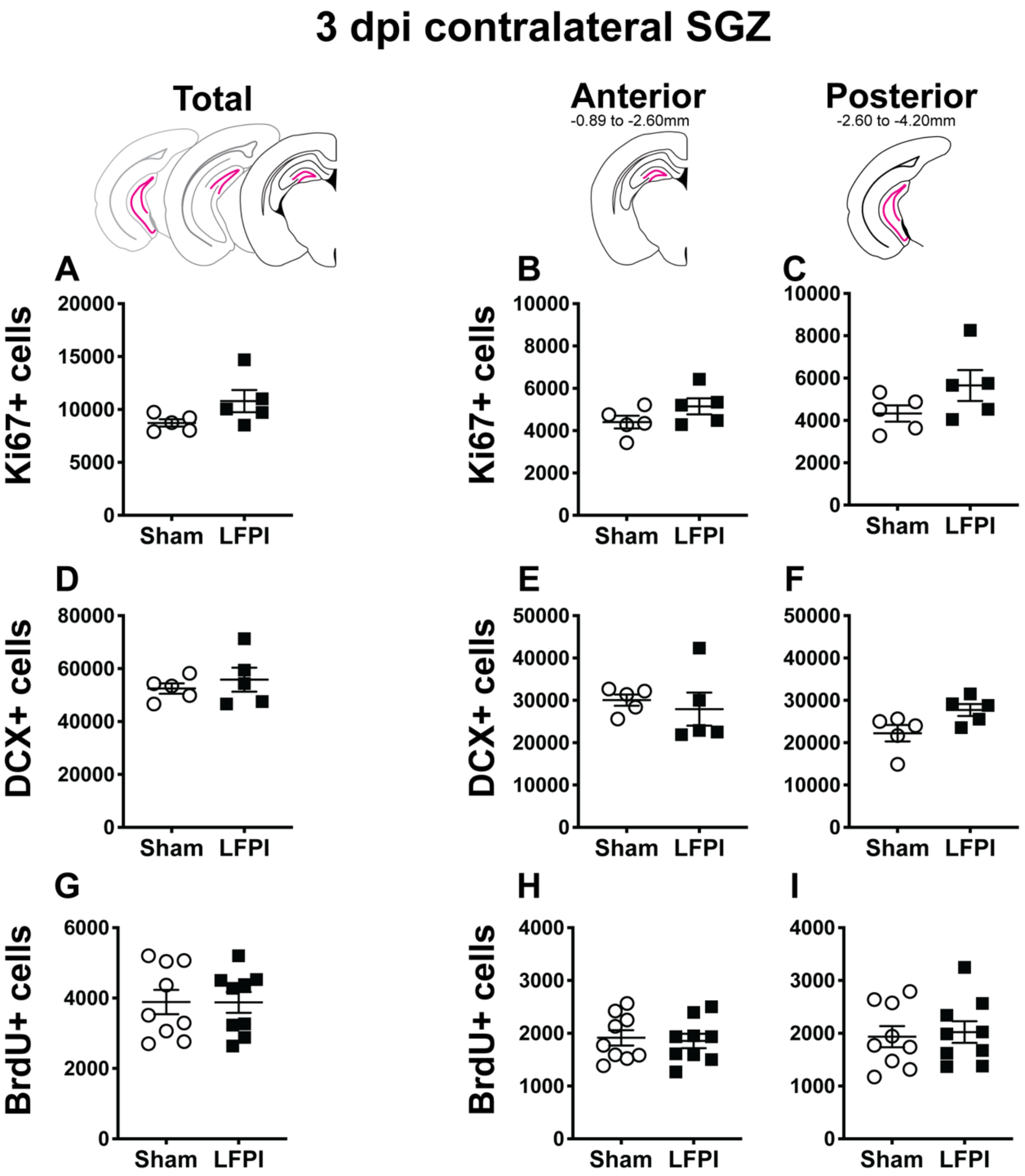

Supplementary Figure 1. Relative to Sham, lateral fluid percussion injury (LFPI) does not change the number of Ki67-immunoreactive (Ki67+), DCX+, or BrdU+ cells in the contralateral mouse subgranular zone (SGZ) or granule cell layer (GCL) 3 days post-injury (dpi).

Pink lines in schematics (top row) indicate these measures were collected in the contralateral SGZ/GCL, while the green lines in the figures in the main text indicate those measures were collected in the ipsilateral SGZ/GCL. Stereological quantification of Ki67+ **(A-C**; Sham n=5, LFPI n=5**)**, DCX+ **(D-F**; Sham n=5, LFPI n=5**)**, and BrdU+ **(G-I**; Sham n=9, LFPI n=9**)** cells in the SGZ

(Ki67, BrdU) and GCL (DCX). Immunopositive cells were quantified across the entire longitudinal axis **(A, D, G)**, and also broken up into anterior **(B, E, H)** and posterior **(C, F, I)** bins, operationally defined as Bregma levels -0.92 to -2.6; and -2.6 to -3.97, respectively.

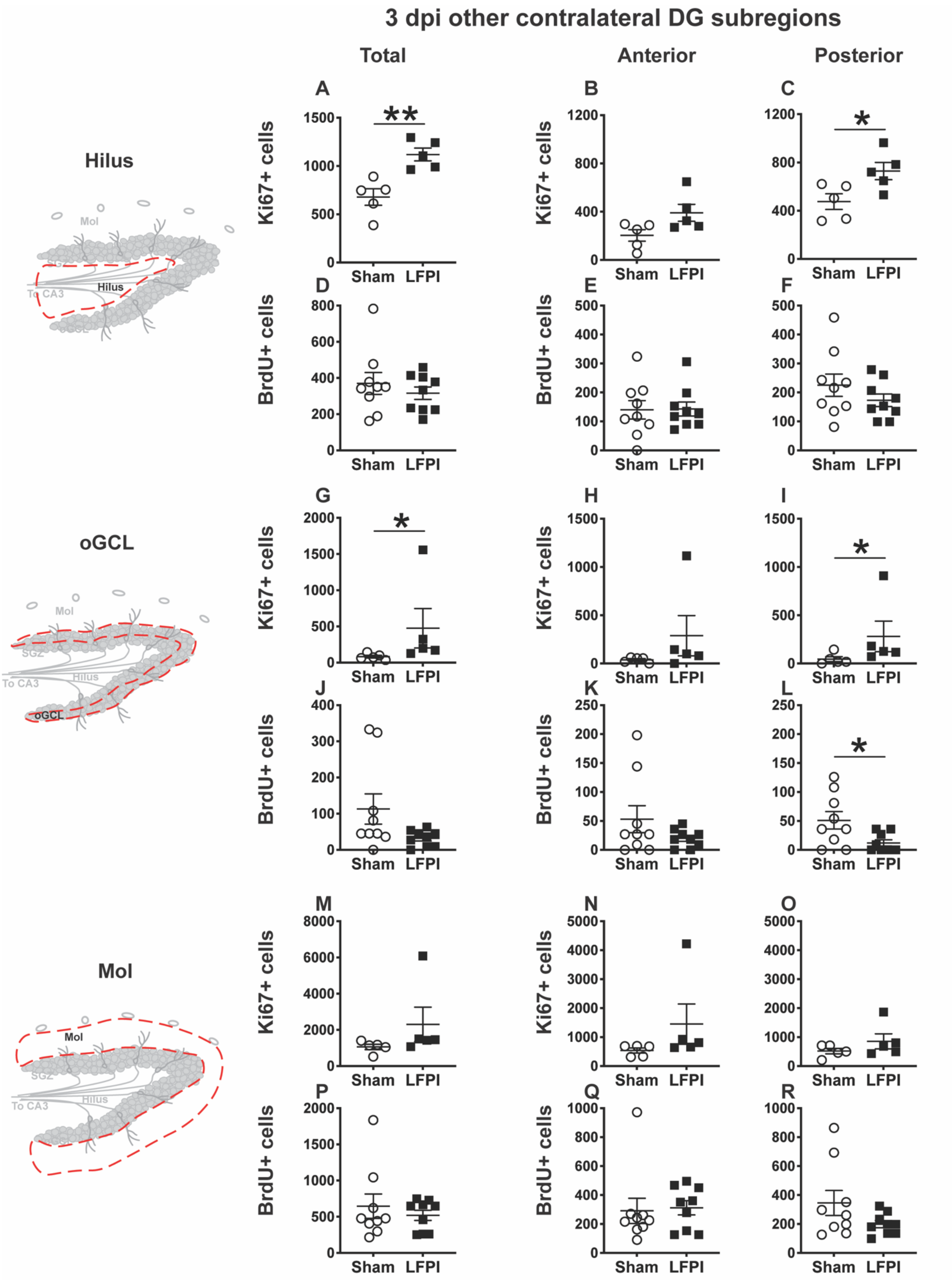
3 dpi other contralateral DG subregions
Total
Anterior
Posterior
Hilus
oGCL
Mol
Ki67+ cells
BrdU+ cells
Sham
LFPI
A
B
C
D
E
F
G
H
I
J
K
L
M
N
O
P
Q
R
**
*
SGZ
Hilus
To CA3
oGCL

Supplementary Figure 2. Relative to Sham treatment, LFPI increases proliferation in some contralateral other DG subregions 3 days post-injury.

Stereological quantification of Ki67+ (**A-C, G-I, M-O**; Sham n=5, LFPI n=5) and BrdU+ (**D-F, J-L, P-R**; Sham n=9, LFPI n=9) cells in the hilus (**A-F**; red dotted line region, top-left schematic), outer granule cell layer (**G-L**; red dotted line region, middle-left schematic), and molecular layer (**M-R**; red dotted line region, bottom-left schematic). Immunopositive cells were quantified across the entire longitudinal axis **(A, D, G, J, M, P)**, and also broken up into anterior **(B, E, H, K, N, Q)** and posterior **(C, F, I, L, O, R)** bins, operationally defined as Bregma levels -0.92 to -2.6; and -2.6 to -3.97, respectively. T-test, *p<0.05, **p<0.01.

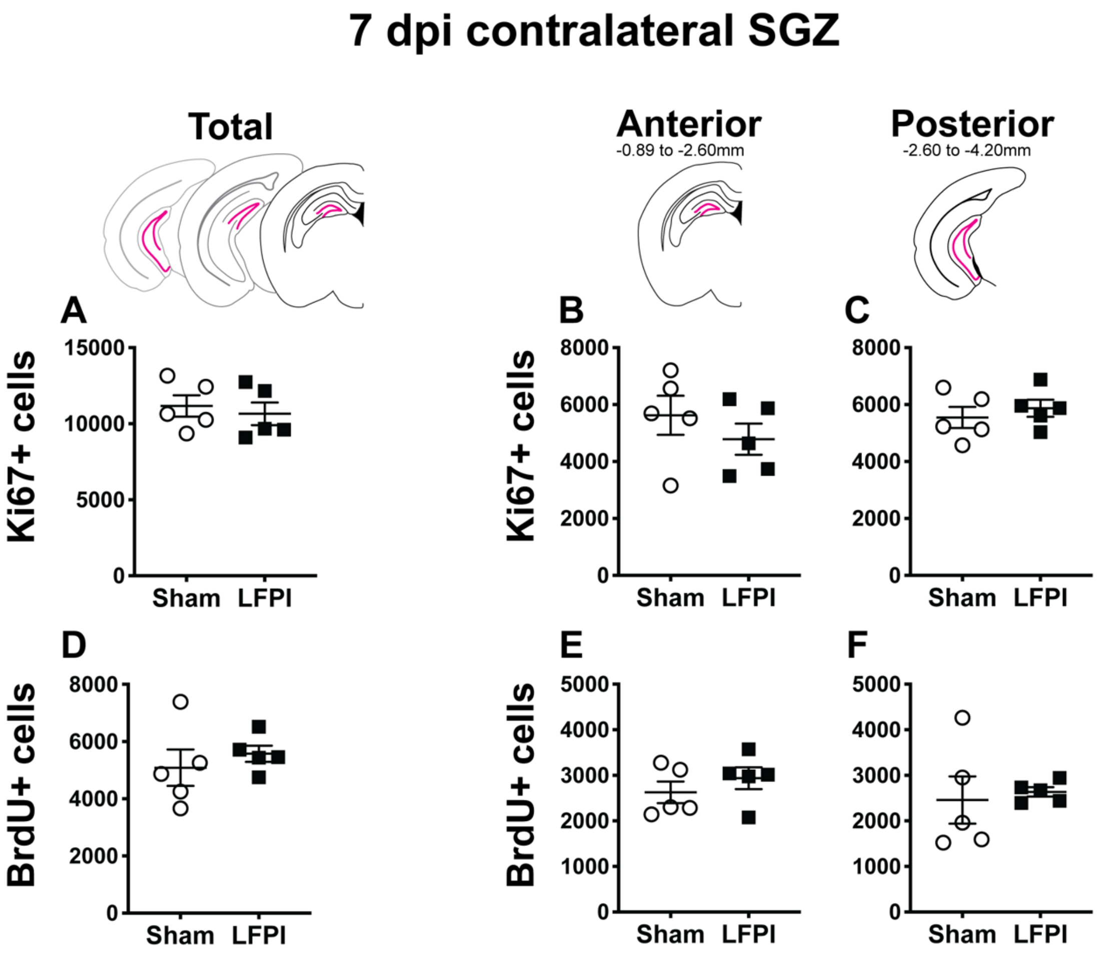

Supplementary Figure 3. LFPI does not affect proliferation and cell survival in the contralateral mouse SGZ 7 days post-injury.

Pink lines in schematics (top row) indicate these measures were collected in the contralateral SGZ/GCL. Stereological quantification of Ki67+ (**A-C**; Sham n=5, LFPI n=5) and BrdU+ (**D-F**; Sham n=5, LFPI n=5) cells in the SGZ (Ki67, BrdU) and GCL (DCX). Immunopositive cells were quantified across the entire longitudinal axis **(A, D)**, and also broken up into anterior **(B, E)** and posterior **(C, F)** bins, operationally defined as Bregma levels -0.92 to -2.6; and -2.6 to -3.97, respectively.

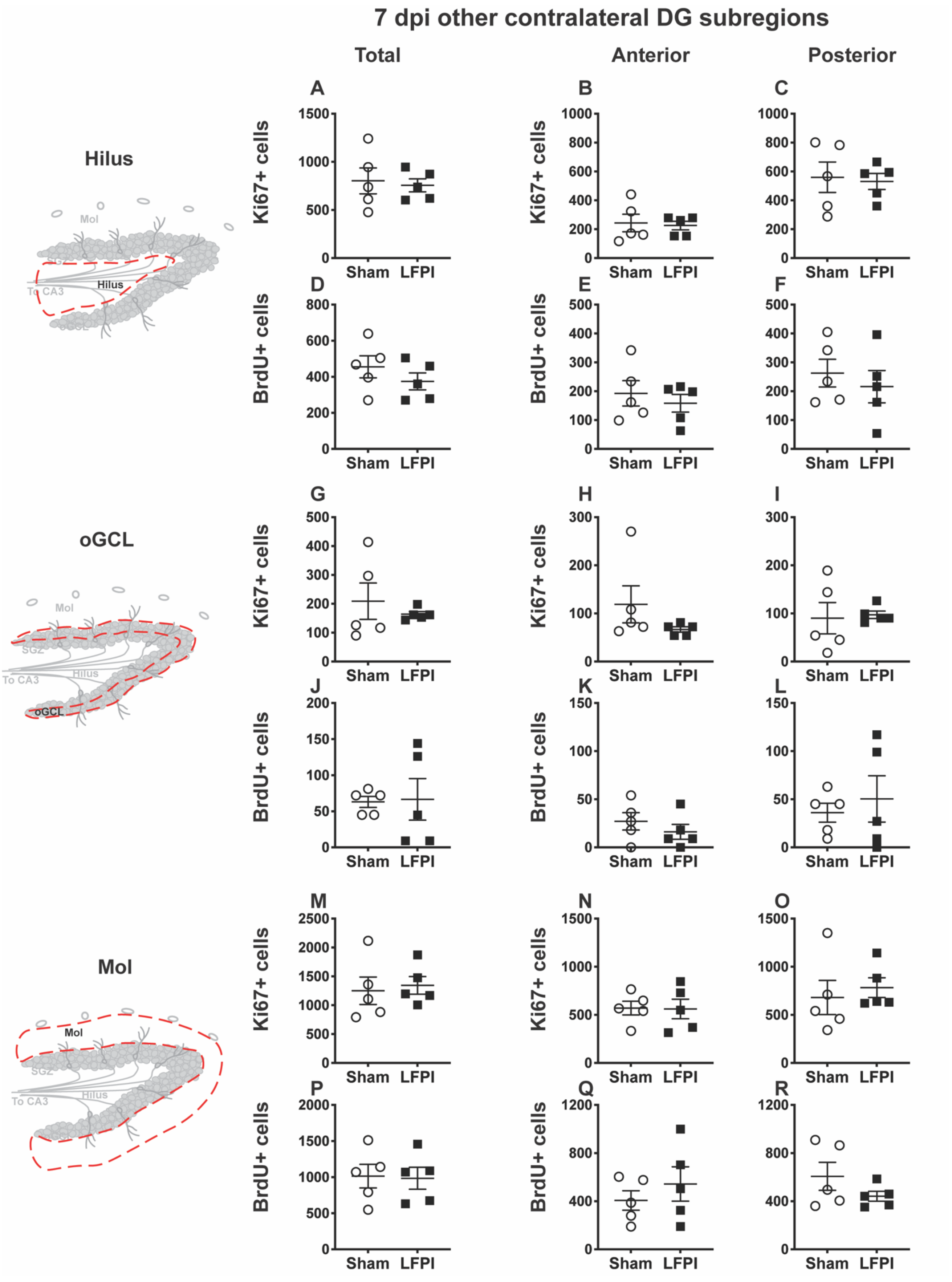
7 dpi other contralateral DG subregions
Total
Anterior
Posterior
Hilus
oGCL
Mol
Ki67+ cells
BrdU+ cells
Sham
LFPI
A
B
C
D
E
F
G
H
I
J
K
L
M
N
O
P
Q
R
SGZ
To CA3
Hilus
oGCL
Mol

Supplementary Figure 4. LFPI does not affect proliferation and intermediate cell survival in other DG subregions 7 days post-injury.

Stereological quantification of Ki67+ (**A-C, G-I, M-O**; Sham n=5, LFPI n=5) and BrdU+ (**D-F, J-L, P-R**; Sham n=5, LFPI n=5) cells in the hilus (**A-F**; red dotted line region, top-left schematic), outer granule cell layer (**G-L**; red dotted line region, middle-left schematic), and molecular layer (**M-R**; red dotted line region, bottom-left schematic). Immunopositive cells were quantified across the entire longitudinal axis **(A, D, G, J, M, P)**, and also broken up into anterior **(B, E, H, K, N, Q)** and posterior **(C, F, I, L, O, R)** bins, operationally defined as Bregma levels -0.92 to -2.6; and -2.6 to -3.97, respectively.

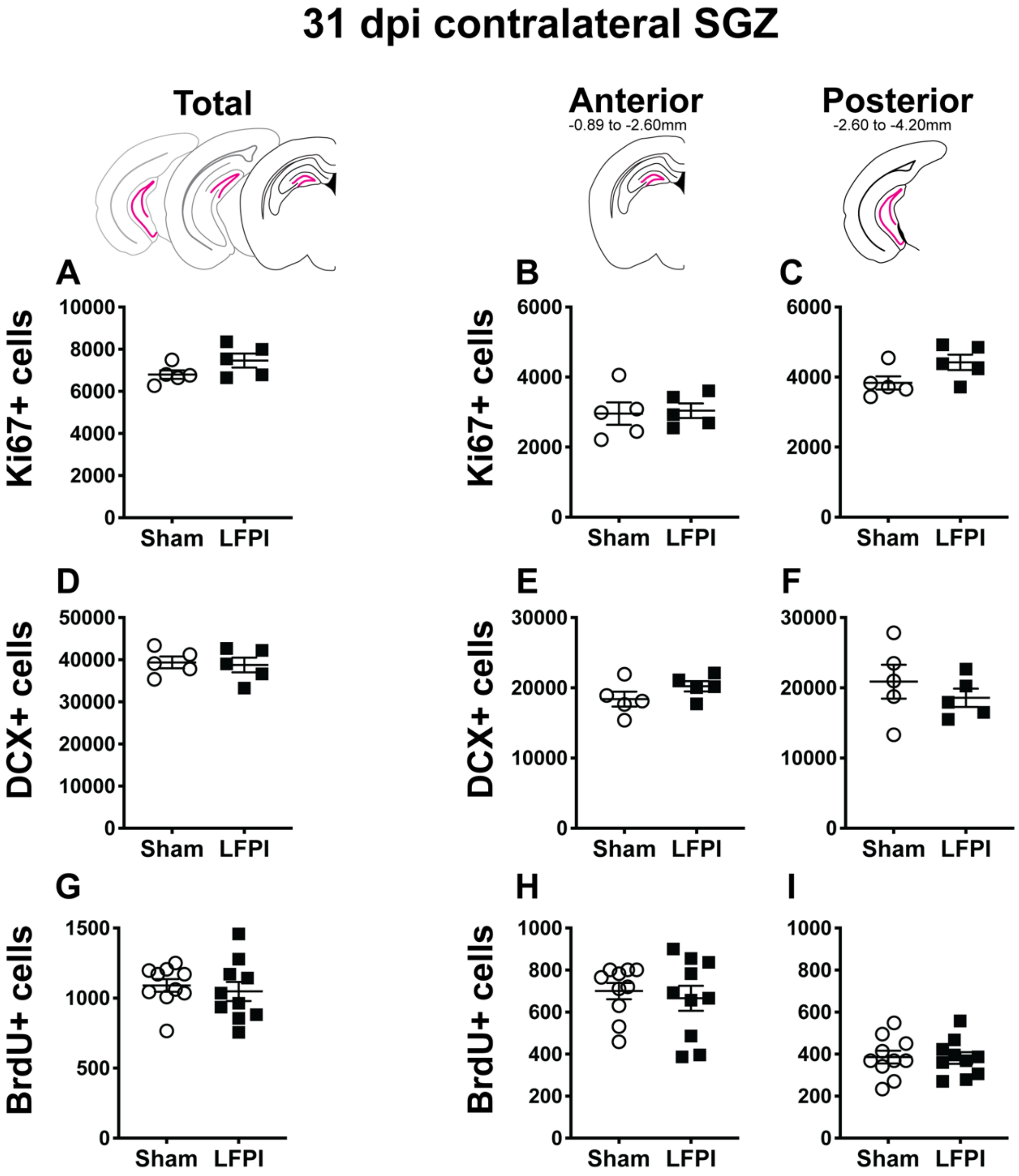

Supplementary Figure 5. LFPI does not affect proliferation, neurogenesis, or long-term cell survival in the contralateral mouse SGZ 31 days post-injury. Pink lines in schematics (top row) indicate these measures were collected in the contralateral SGZ/GCL.

Pink lines in schematics (top row) indicate these measures were collected in the contralateral SGZ/GCL. Stereological quantification of Ki67+ (**A-C**; Sham n=5, LFPI n=5), DCX+ (**D-F**; Sham n=5, LFPI n=5), and BrdU+ (**G-I**; Sham n=10, LFPI n=10) cells in the SGZ (Ki67, BrdU) and GCL (DCX). Immunopositive cells were quantified across the entire longitudinal axis **(A, D, G)**, and also broken up into anterior **(B, E, H)** and posterior **(C, F, I)** bins, operationally defined as Bregma levels -0.92 to -2.6; and -2.6 to -3.97, respectively.

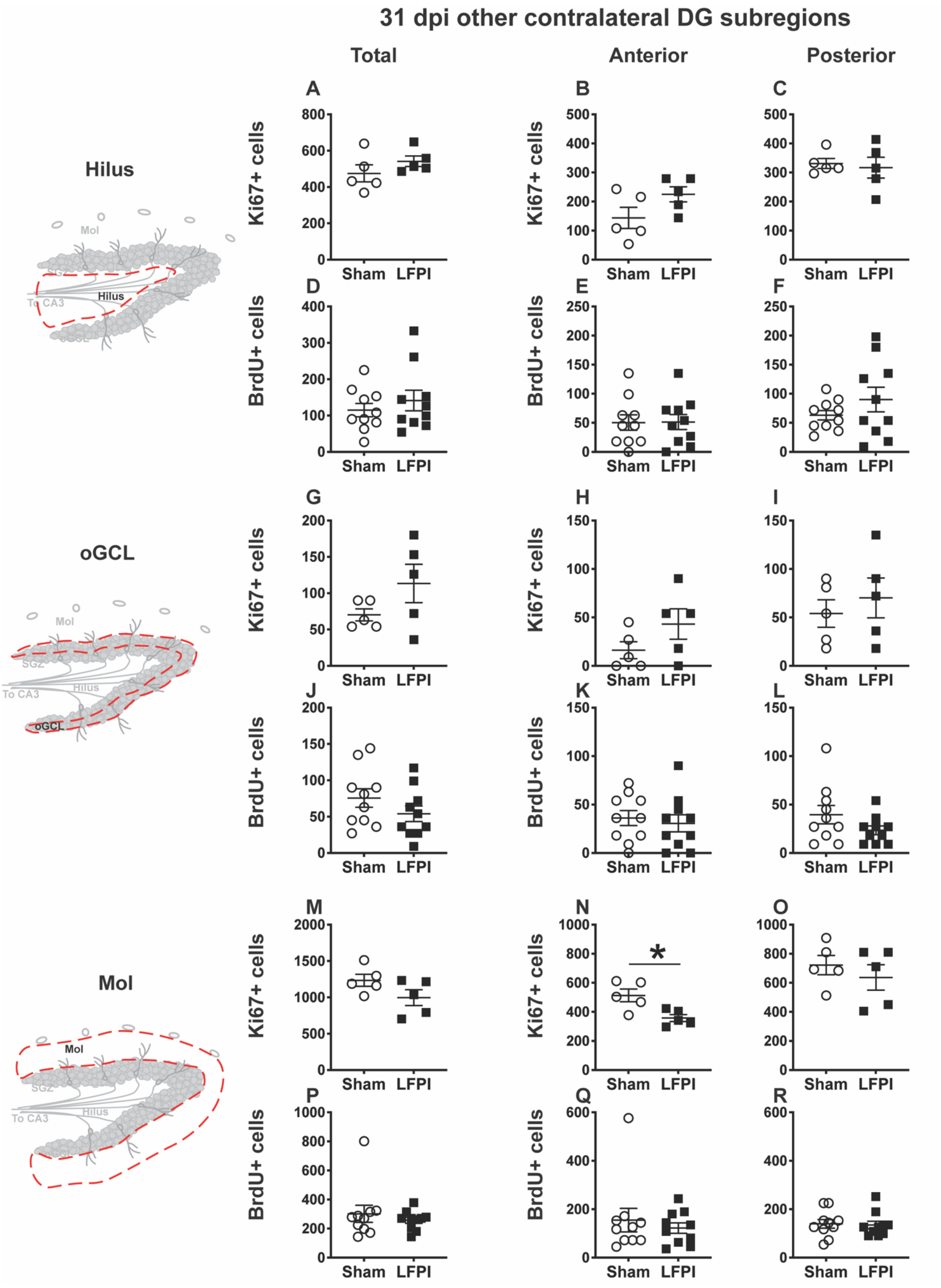
31 dpi other contralateral DG subregions
Total
Anterior
Posterior
Hilus
oGCL
Mol
Ki67+ cells
BrdU+ cells
Sham
LFPI
A
B
C
D
E
F
G
H
I
J
K
L
M
N
O
P
Q
R
SGZ
To CA3
Hilus
oGCL
*

Supplementary Figure 6. LFPI does not affect proliferation or long-term cell survival in other contralateral DG subregions 31 days post-injury.
Stereological quantification of Ki67+ (**A-C, G-I, M-O**; Sham n=5, LFPI n=5) and BrdU+ (**D-F, J-L, P-R**; Sham n=10, LFPI n=10) cells in the hilus (**A-F**; red dotted line region, top-left schematic), outer granule cell layer (**G-L**; red dotted line region, middle-left schematic), and molecular layer (**M-R**; red dotted line region, bottom-left schematic). Immunopositive cells were quantified across the entire longitudinal axis (**A, D, G, J, M, P**), and also broken up into anterior (**B, E, H, K, N, Q**) and posterior (**C, F, I, L, O, R**) bins, operationally defined as Bregma levels -0.92 to -2.6; and -2.6 to -3.97, respectively.

Supplementary Table 1. Statistical results for cell quantification in the ipsilateral hemisphere.

3 DPI - IPSILATERAL

Marker	Region	Totals				Anterior				Posterior			
**Ki67**	**SGZ**	**Fig. 2A**				**Fig. 2B**				**Fig. 2C**			
		**Sham**		**LFPI**		**Sham**		**LFPI**		**Sham**		**LFPI**	
		Mean +/- SEM	n	Mean +/- SEM	n	Mean +/- SEM	n	Mean +/- SEM	n	Mean +/- SEM	n	Mean +/- SEM	n
		9661 +/- 558.1	9	13922 +/- 843.1	9	4119 +/- 284.7	9	5679 +/- 339.3	9	5542 +/- 349.3	9	8243 +/- 767.8	9
		t-test (p-value)				t-test (p-value)				t-test (p-value)			
		0.0007				0.0028				0.0056			
	**Hilus**	**Fig. 3A**				**Fig. 3B**				**Fig. 3C**			
		**Sham**		**LFPI**		**Sham**		**LFPI**		**Sham**		**LFPI**	
		Mean +/- SEM	n	Mean +/- SEM	n	Mean +/- SEM	n	Mean +/- SEM	n	Mean +/- SEM	n	Mean +/- SEM	n
		1023 +/- 195	9	2417 +/- 352.1	9	306 +/- 38.65	9	621 +/- 100.8	9	717 +/- 172	9	1796 +/- 280.3	9
		Mann-Whitney (p-value)				t-test (p-value)				Mann-Whitney (p-value)			
		0.0028				0.0101				0.0021			
	**oGCL**	**Fig. 3G**				**Fig. 3H**				**Fig. 3I**			
		**Sham**		**LFPI**		**Sham**		**LFPI**		**Sham**		**LFPI**	
		Mean +/- SEM	n	Mean +/- SEM	n	Mean +/- SEM	n	Mean +/- SEM	n	Mean +/- SEM	n	Mean +/- SEM	n
		373 +/- 166.6	9	1406 +/- 374.3	9	135 +/- 48	9	485 +/- 134.9	9	237 +/- 125.9	9	921 +/- 286.1	9
		Mann-Whitney (p-value)				Mann-Whitney (p-value)				Mann-Whitney (p-value)			
		0.0099				0.0422				0.037			
	**Mol**	**Fig. 3M**				**Fig. 3N**				**Fig. 3O**			
		**Sham**		**LFPI**		**Sham**		**LFPI**		**Sham**		**LFPI**	
		Mean +/- SEM	n	Mean +/- SEM	n	Mean +/- SEM	n	Mean +/- SEM	n	Mean +/- SEM	n	Mean +/- SEM	n
		2642 +/- 854.9	9	8916 +/- 1956	9	796 +/- 190	9	2777 +/- 581.3	9	1846 +/- 672.8	9	6139 +/- 1418	9
		Mann-Whitney (p-value)				Mann-Whitney (p-value)				Mann-Whitney (p-value)			
		0.0056				0.0074				0.0078			
**DCX**	**SGZ + GCL**	**Fig. 2D**				**Fig. 2E**				**Fig. 2F**			
		**Sham**		**LFPI**		**Sham**		**LFPI**		**Sham**		**LFPI**	
		Mean +/- SEM	n	Mean +/- SEM	n	Mean +/- SEM	n	Mean +/- SEM	n	Mean +/- SEM	n	Mean +/- SEM	n
		52329 +/- 4968	5	54905 +/- 5013	5	26217 +/- 3198	5	25861 +/- 4479	5	25927 +/- 2358	5	28919 +/- 1118	5
		t-test (p-value)				t-test (p-value)				t-test (p-value)			
		0.7246				0.95				0.2848			
**BrdU**	**SGZ**	**Fig. 2G**				**Fig. 2H**				**Fig. 2I**			
		**Sham**		**LFPI**		**Sham**		**LFPI**		**Sham**		**LFPI**	
		Mean +/- SEM	n	Mean +/- SEM	n	Mean +/- SEM	n	Mean +/- SEM	n	Mean +/- SEM	n	Mean +/- SEM	n
		3927 +/- 292.4	9	4969 +/- 507	9	1961 +/- 187.4	9	2173 +/- 189.3	9	1795 +/- 149.8	9	2492 +/- 386.3	9
		t-test (p-value)				t-test (p-value)				Mann-Whitney (p-value)			
		0.094				0.4377				0.1359			
	**Hilus**	**Fig. 3D**				**Fig. 3E**				**Fig. 3F**			
		**Sham**		**LFPI**		**Sham**		**LFPI**		**Sham**		**LFPI**	
		Mean +/- SEM	n	Mean +/- SEM	n	Mean +/- SEM	n	Mean +/- SEM	n	Mean +/- SEM	n	Mean +/- SEM	n
		414 +/- 75.64	9	970 +/- 104.1	9	149 +/- 31.32	9	281 +/- 47.31	9	223 +/- 35.93	9	562 +/- 84.92	9
		t-test (p-value)				t-test (p-value)				t-test (p-value)			
		0.0005				0.0335				0.002			
	**oGCL**	**Fig. 3J**				**Fig. 3K**				**Fig. 3L**			
		**Sham**		**LFPI**		**Sham**		**LFPI**		**Sham**		**LFPI**	
		Mean +/- SEM	n	Mean +/- SEM	n	Mean +/- SEM	n	Mean +/- SEM	n	Mean +/- SEM	n	Mean +/- SEM	n
		77 +/- 16.3	9	139 +/- 26.29	9	22 +/- 9.5	9	30 +/- 8.485	9	54 +/- 9.367	9	95 +/- 17.56	9
		t-test (p-value)				Mann-Whitney (p-value)				Mann-Whitney (p-value)			
		0.0623				0.2581				0.1629			
	**Mol**	**Fig. 3P**				**Fig. 3Q**				**Fig. 3R**			
		**Sham**		**LFPI**		**Sham**		**LFPI**		**Sham**		**LFPI**	
		Mean +/- SEM	n	Mean +/- SEM	n	Mean +/- SEM	n	Mean +/- SEM	n	Mean +/- SEM	n	Mean +/- SEM	n
		675 +/- 181.5	9	1749 +/- 273.3	9	256 +/- 53.1	9	462 +/- 67.45	9	307 +/- 73.63	9	1127 +/- 183.3	9
		Mann-Whitney (p-value)				t-test (p-value)				t-test (p-value)			
		0.0025				0.0289				0.0008			

7 DPI - IPSILATERAL

Marker	Region	Totals				Anterior				Posterior			
Ki67	SGZ	**Fig. 4A**				**Fig. 4B**				**Fig. 4C**			
		**Sham**		**LFPI**		**Sham**		**LFPI**		**Sham**		**LFPI**	
		Mean +/- SEM	n	Mean +/- SEM	n	Mean +/- SEM	n	Mean +/- SEM	n	Mean +/- SEM	n	Mean +/- SEM	n
		12537 +/- 1291	5	13815 +/- 912.3	5	5611 +/- 791.1	5	6707 +/- 564.8	5	6926 +/- 587.3	5	7108 +/- 473.1	5
		t-test (p-value)				t-test (p-value)				t-test (p-value)			
		0.4421				0.2921				0.8156			
	Hilus	**Fig. 5A**				**Fig. 5B**				**Fig. 5C**			
		**Sham**		**LFPI**		**Sham**		**LFPI**		**Sham**		**LFPI**	
		Mean +/- SEM	n	Mean +/- SEM	n	Mean +/- SEM	n	Mean +/- SEM	n	Mean +/- SEM	n	Mean +/- SEM	n
		1031 +/- 158.8	5	873 +/- 28.8	5	252 +/- 25.14	5	316.8	5	779.4 +/- 136.9	5	556.2 +/- 42.06	5
		t-test (p-value)				Mann-Whitney (p-value)				t-test (p-value)			
		0.3551				0.0873				0.1577			
	oGCL	**Fig. 5G**				**Fig. 5H**				**Fig. 5I**			
		**Sham**		**LFPI**		**Sham**		**LFPI**		**Sham**		**LFPI**	
		Mean +/- SEM	n	Mean +/- SEM	n	Mean +/- SEM	n	Mean +/- SEM	n	Mean +/- SEM	n	Mean +/- SEM	n
		180 +/- 36.34	5	345.6 +/- 81.13	5	45 +/- 12.73	5	84.60 +/- 17.91	5	135 +/- 26.24	5	261 +/- 63.64	5
		t-test (p-value)				t-test (p-value)				t-test (p-value)			
		0.0995				0.1092				0.1046			
	Mol	**Fig. 5M**				**Fig. 5N**				**Fig. 5O**			
		**Sham**		**LFPI**		**Sham**		**LFPI**		**Sham**		**LFPI**	
		Mean +/- SEM	n	Mean +/- SEM	n	Mean +/- SEM	n	Mean +/- SEM	n	Mean +/- SEM	n	Mean +/- SEM	n
		1678 +/- 309	5	1071 +/- 245.9	5	693 +/- 82.09	5	446.4 +/- 74.47	5	984.6 +/- 228.2	5	624.6 +/- 174.8	5
		t-test (p-value)				t-test (p-value)				t-test (p-value)			
		0.1631				0.0567				0.2458			
DCX	SGZ + GCL	**Fig. 4D**				**Fig. 4E**				**Fig. 4F**			
		**Sham**		**LFPI**		**Sham**		**LFPI**		**Sham**		**LFPI**	
		Mean +/- SEM	n	Mean +/- SEM	n	Mean +/- SEM	n	Mean +/- SEM	n	Mean +/- SEM	n	Mean +/- SEM	n
		47142 +/- 2746	5	63115 +/- 3741	5	23027 +/- 2979	5	29810 +/- 996.8	5	23969 +/- 1423	5	33055 +/- 3186	5
		t-test (p-value)				t-test (p-value)				t-test (p-value)			
		0.0088				0.0629				0.0314			
BrdU	SGZ	**Fig. 4G**				**Fig. 4H**				**Fig. 4I**			
		**Sham**		**LFPI**		**Sham**		**LFPI**		**Sham**		**LFPI**	
		Mean +/- SEM	n	Mean +/- SEM	n	Mean +/- SEM	n	Mean +/- SEM	n	Mean +/- SEM	n	Mean +/- SEM	n
		5683 +/- 1000	5	6340 +/- 457.8	5	2839 +/- 550.4	5	3343 +/- 183.6	5	2844 +/- 481.2	5	2997 +/- 320.4	5
		Mann-Whitney (p-value)				Mann-Whitney (p-value)				t-test (p-value)			
		0.1508				0.1508				0.798			
	Hilus	**Fig. 5D**				**Fig. 5E**				**Fig. 5F**			
		**Sham**		**LFPI**		**Sham**		**LFPI**		**Sham**		**LFPI**	
		Mean +/- SEM	n	Mean +/- SEM	n	Mean +/- SEM	n	Mean +/- SEM	n	Mean +/- SEM	n	Mean +/- SEM	n
		504 +/- 30.65	5	856.8 +/- 190.9	5	138.6 +/- 31.64	5	183.6 +/- 58.71	5	365.4 +/- 20.64	5	673.2 +/- 142.7	5
		t-test (p-value)				t-test (p-value)				t-test (p-value)			
		0.1055				0.5189				0.0653			
	oGCL	**Fig. 5J**				**Fig. 5K**				**Fig. 5L**			
		**Sham**		**LFPI**		**Sham**		**LFPI**		**Sham**		**LFPI**	
		Mean +/- SEM	n	Mean +/- SEM	n	Mean +/- SEM	n	Mean +/- SEM	n	Mean +/- SEM	n	Mean +/- SEM	n
		122.4 +/- 20.24	5	142.2 +/- 44.22	5	48.60 +/- 20.04	5	37.8 +/- 14.34	5	73.8 +/- 10.02	5	104.4 +/- 32.02	
		t-test (p-value)				t-test (p-value)				t-test (p-value)			
		0.6946				0.6728				0.3884			
	Mol	**Fig. 5P**				**Fig. 5Q**				**Fig. 5R**			
		**Sham**		**LFPI**		**Sham**		**LFPI**		**Sham**		**LFPI**	
		Mean +/- SEM	n	Mean +/- SEM	n	Mean +/- SEM	n	Mean +/- SEM	n	Mean +/- SEM	n	Mean +/- SEM	n
		1771 +/- 531.4	5	2050 +/- 534.8	5	795.6 +/- 385.1	5	595.8 +/- 226.8	5	975.6 +/- 189	5	1454 +/- 477.4	5
		t-test (p-value)				Mann-Whitney (p-value)				t-test (p-value)			
		0.7209				0.8413				0.3783			

31 DPI - IPSILATERAL

Marker	Region	Totals				Anterior				Posterior			
**Ki67**	**SGZ**	**Fig. 6A**				**Fig. 6B**				**Fig. 6C**			
		**Sham**		**LFPI**		**Sham**		**LFPI**		**Sham**		**LFPI**	
		Mean +/- SEM	n	Mean +/- SEM	n	Mean +/- SEM	n	Mean +/- SEM	n	Mean +/- SEM	n	Mean +/- SEM	n
		6323 +/- 465.6	5	6134 +/- 485.9	5	2925 +/- 150.4	5	2434 +/- 218	5	3398 +/- 472.2	5	3701 +/- 330.5	5
		t-test (p-value)				t-test (p-value)				t-test (p-value)			
		0.786				0.1006				0.6141			
	**Hilus**	**Fig. 7A**				**Fig. 7B**				**Fig. 7C**			
		**Sham**		**LFPI**		**Sham**		**LFPI**		**Sham**		**LFPI**	
		Mean +/- SEM	n	Mean +/- SEM	n	Mean +/- SEM	n	Mean +/- SEM	n	Mean +/- SEM	n	Mean +/- SEM	n
		639 +/- 118.5	5	433.8 +/- 59.04	5	217.8 +/- 66.65	5	118.8 +/- 26.61	5	421.2 +/- 64.92	5	315 +/- 54.52	5
		t-test (p-value)				t-test (p-value)				t-test (p-value)			
		0.1597				0.2051				0.2457			
	**oGCL**	**Fig. 7G**				**Fig. 7H**				**Fig. 7I**			
		**Sham**		**LFPI**		**Sham**		**LFPI**		**Sham**		**LFPI**	
		Mean +/- SEM	n	Mean +/- SEM	n	Mean +/- SEM	n	Mean +/- SEM	n	Mean +/- SEM	n	Mean +/- SEM	n
		106.2 +/- 31.1	5	144 +/- 60.91	5	3.6 +/- 3.6	5	21.6 +/- 10.87	5	102.6 +/- 29.11	5	122.4 +/- 64.56	5
		t-test (p-value)				Mann-Whitney (p-value)				t-test (p-value)			
		0.5956				0.1667				0.7869			
	**Mol**	**Fig. 7M**				**Fig. 7N**				**Fig. 7O**			
		**Sham**		**LFPI**		**Sham**		**LFPI**		**Sham**		**LFPI**	
		Mean +/- SEM	n	Mean +/- SEM	n	Mean +/- SEM	n	Mean +/- SEM	n	Mean +/- SEM	n	Mean +/- SEM	n
		1136 +/- 117.8	5	986.4 +/- 176.7	5	525.6 +/- 88.53	5	383.4 +/- 83.1	5	610.2 +/- 33.36	5	603 +/- 101.1	5
		t-test (p-value)				t-test (p-value)				t-test (p-value)			
		0.5018				0.2753				0.9478			
**DCX**	**SGZ + GCL**	**Fig. 6D**				**Fig. 6E**				**Fig. 6F**			
		**Sham**		**LFPI**		**Sham**		**LFPI**		**Sham**		**LFPI**	
		Mean +/- SEM	n	Mean +/- SEM	n	Mean +/- SEM	n	Mean +/- SEM	n	Mean +/- SEM	n	Mean +/- SEM	n
		42261 +/- 3818	5	39743 +/- 1433	5	19915 +/- 1951	5	20212 +/- 760.6	5	22306 +/- 2788	5	19525 +/- 1674	5
		t-test (p-value)				t-test (p-value)				t-test (p-value)			
		0.5541				0.8907				0.4174			
**BrdU**	**SGZ**	**Fig. 6G**				**Fig. 6H**				**Fig. 6I**			
		**Sham**		**LFPI**		**Sham**		**LFPI**		**Sham**		**LFPI**	
		Mean +/- SEM	n	Mean +/- SEM	n	Mean +/- SEM	n	Mean +/- SEM	n	Mean +/- SEM	n	Mean +/- SEM	n
		1179 +/- 84.84	10	1726 +/- 92.11	10	750.6 +/- 68.39	10	972 +/- 64.89	10	422.1 +/- 34.69	10	748.8 +/- 52.34	10
		t-test (p-value)				t-test (p-value)				t-test (p-value)			
		0.0004				0.0305				<0.0001			
	**Hilus**	**Fig. 7D**				**Fig. 7E**				**Fig. 7F**			
		**Sham**		**LFPI**		**Sham**		**LFPI**		**Sham**		**LFPI**	
		Mean +/- SEM	n	Mean +/- SEM	n	Mean +/- SEM	n	Mean +/- SEM	n	Mean +/- SEM	n	Mean +/- SEM	n
		146.7 +/- 23.43	10	828 +/- 93.35	10	53.1 +/- 12.4	10	298.8 +/- 45.02	10	91.8 +/- 16.31	10	526.5 +/- 54.68	10
		Mann-Whitney (p-value)				Mann-Whitney (p-value)				t-test (p-value)			
		<0.0001				<0.0001				<0.0001			
	**oGCL**	**Fig. 7J**				**Fig. 7K**				**Fig. 7L**			
		**Sham**		**LFPI**		**Sham**		**LFPI**		**Sham**		**LFPI**	
		Mean +/- SEM	n	Mean +/- SEM	n	Mean +/- SEM	n	Mean +/- SEM	n	Mean +/- SEM	n	Mean +/- SEM	n
		92.7 +/- 24.56	10	162 +/- 15.76	10	43.2 +/- 13.34	10	67.5 +/- 15.13	10	49.5 +/- 13.57	10	92.7 +/- 11.47	10
		Mann-Whitney (p-value)				Mann-Whitney (p-value)				t-test (p-value)			
		0.005				0.1675				0.0257			
	**Mol**	**Fig. 7P**				**Fig. 7Q**				**Fig. 7R**			
		**Sham**		**LFPI**		**Sham**		**LFPI**		**Sham**		**LFPI**	
		Mean +/- SEM	n	Mean +/- SEM	n	Mean +/- SEM	n	Mean +/- SEM	n	Mean +/- SEM	n	Mean +/- SEM	n
		421.2 +/- 102.2	10	1382 +/- 168.1	10	142.2 +/- 31.63	10	440.1 +/- 105.4	10	260.1 +/- 75.82	10	934.2 +/- 138.9	10
		Mann-Whitney (p-value)				Mann-Whitney (p-value)				Mann-Whitney (p-value)			
		0.0027				0.003				0.0027			

Supplementary Table 2. Statistical results for cell quantification in the contralateral hemisphere.

3 DPI - CONTRALATERAL													
Marker	Region	Totals				Anterior				Posterior			
Ki67	SGZ	**Fig. S1A**				**Fig. S1B**				**Fig. S1C**			
		**Sham**		**LFPI**		**Sham**		**LFPI**		**Sham**		**LFPI**	
		Mean +/- SEM	n	Mean +/- SEM	n	Mean +/- SEM	n	Mean +/- SEM	n	Mean +/- SEM	n	Mean +/- SEM	n
		8732 +/- 348.2	5	10798 +/- 1050		4405 +/- 296.4	5	5148 +/- 378.5	5	4327 +/- 382.4	5	5650 +/- 729.9	5
		t-test (p-value)				t-test (p-value)				t-test (p-value)			
		0.0986				0.1606				0.147			
	Hilus	**Fig. S2A**				**Fig. S2B**				**Fig. S2C**			
		**Sham**		**LFPI**		**Sham**		**LFPI**		**Sham**		**LFPI**	
		Mean +/- SEM	n	Mean +/- SEM	n	Mean +/- SEM	n	Mean +/- SEM	n	Mean +/- SEM	n	Mean +/- SEM	n
		678.6 +/- 85.22	5	1120 +/- 66.17	5	203.4 +/- 48.27	5	390.6 +/- 70.5	5	475.2 +/- 64.92	5	729 +/- 71.94	5
		t-test (p-value)				t-test (p-value)				t-test (p-value)			
		0.0035				0.0598				0.0307			
	oGCL	**Fig. S2G**				**Fig. S2H**				**Fig. S2I**			
		**Sham**		**LFPI**		**Sham**		**LFPI**		**Sham**		**LFPI**	
		Mean +/- SEM	n	Mean +/- SEM	n	Mean +/- SEM	n	Mean +/- SEM	n	Mean +/- SEM	n	Mean +/- SEM	n
		82.8 +/- 18.31	5	475.2 +/- 272.4	5	37.8 +/- 12.21	5	288 +/- 208.3	5	45 +/- 25.77	5	280.8 +/- 158	5
		Mann-Whitney (p-value)				Mann-Whitney (p-value)				Mann-Whitney (p-value)			
		0.0159				0.1111				0.0476			
	Mol	**Fig. S2M**				**Fig. S2N**				**Fig. S2O**			
		**Sham**		**LFPI**		**Sham**		**LFPI**		**Sham**		**LFPI**	
		Mean +/- SEM	n	Mean +/- SEM	n	Mean +/- SEM	n	Mean +/- SEM	n	Mean +/- SEM	n	Mean +/- SEM	n
		1058 +/- 146.5	5	2306 +/- 947.6	5	540 +/- 88.27	5	1451 +/- 694.6	5	518.4 +/- 93.51	5	855 +/- 260.5	5
		Mann-Whitney (p-value)				Mann-Whitney (p-value)				Mann-Whitney (p-value)			
		0.0556				0.2222				0.5159			
DCX	SGZ + GCL	**Fig. S1D**				**Fig. S1E**				**Fig. S1F**			
		**Sham**		**LFPI**		**Sham**		**LFPI**		**Sham**		**LFPI**	
		Mean +/- SEM	n	Mean +/- SEM	n	Mean +/- SEM	n	Mean +/- SEM	n	Mean +/- SEM	n	Mean +/- SEM	n
		52472 +/- 1981	5	55843 +/- 4513	5	30051 +/- 1332	5	27936 +/- 3899	5	22250 +/- 1958	5	27720 +/- 1405	5
		t-test (p-value)				t-test (p-value)				t-test (p-value)			
		0.5133				0.6216				0.0529			
BrdU	SGZ	**Fig. S1G**				**Fig. S1H**				**Fig. S1I**			
		**Sham**		**LFPI**		**Sham**		**LFPI**		**Sham**		**LFPI**	
		Mean +/- SEM	n	Mean +/- SEM	n	Mean +/- SEM	n	Mean +/- SEM	n	Mean +/- SEM	n	Mean +/- SEM	n
		3889 +/- 344.2	9	3878 +/- 295.8	9	1914 +/- 144.7	9	1855 +/- 135.8	9	1935 +/- 200	9	2023 +/- 205.1	9
		t-test (p-value)				t-test (p-value)				t-test (p-value)			
		0.981				0.77				0.7627			
	Hilus	**Fig. S2D**				**Fig. S2E**				**Fig. S2F**			
		**Sham**		**LFPI**		**Sham**		**LFPI**		**Sham**		**LFPI**	
		Mean +/- SEM	n	Mean +/- SEM	n	Mean +/- SEM	n	Mean +/- SEM	n	Mean +/- SEM	n	Mean +/- SEM	n
		370 +/- 60.66	9	316 +/- 34.64	9	140 +/- 31.89	9	143 +/- 23.97	9	225 +/- 38.54	9	173 +/- 21.67	9
		t-test (p-value)				t-test (p-value)				t-test (p-value)			
		0.4508				0.941				0.2567			
	oGCL	**Fig. S2J**				**Fig. S2K**				**Fig. S2L**			
		**Sham**		**LFPI**		**Sham**		**LFPI**		**Sham**		**LFPI**	
		Mean +/- SEM	n	Mean +/- SEM	n	Mean +/- SEM	n	Mean +/- SEM	n	Mean +/- SEM	n	Mean +/- SEM	n
		113 +/- 41.92	9	32 +/- 7.365	9	53 +/- 23.26	9	20 +/- 5.148	9	51 +/- 15.15	9	12 +/- 5.408	9
		Mann-Whitney (p-value)				Mann-Whitney (p-value)				Mann-Whitney (p-value)			
		0.1051				0.4685				0.042			
	Mol	**Fig. S2P**				**Fig. S2Q**				**Fig. S2R**			
		**Sham**		**LFPI**		**Sham**		**LFPI**		**Sham**		**LFPI**	
		Mean +/- SEM	n	Mean +/- SEM	n	Mean +/- SEM	n	Mean +/- SEM	n	Mean +/- SEM	n	Mean +/- SEM	n
		646 +/- 168.3	9	519 +/- 70.61	9	291 +/- 87.14	9	312 +/- 49.48	9	345 +/- 86.66	9	199 +/- 24.53	9
		Mann-Whitney (p-value)				Mann-Whitney (p-value)				Mann-Whitney (p-value)			
		0.9144				0.3735				0.2659			

7 DPI - CONTRALATERAL

Marker	Region	Totals				Anterior				Posterior			
Ki67	SGZ	**Fig. S3A**				**Fig. S3B**				**Fig. S3C**			
		**Sham**		**LFPI**		**Sham**		**LFPI**		**Sham**		**LFPI**	
		Mean +/- SEM	n	Mean +/- SEM	n	Mean +/- SEM	n	Mean +/- SEM	n	Mean +/- SEM	n	Mean +/- SEM	n
		11169 +/- 705.3	5	10654 +/- 744.5	5	5625 +/- 687.5	5	4784 +/- 545.4	5	5544 +/- 370.4	5	5870 +/- 299.4	5
		t-test (p-value)				t-test (p-value)				t-test (p-value)			
		0.6292				0.3662				0.5133			
	hilus	**Fig. S4A**				**Fig. S4B**				**Fig. S4C**			
		**Sham**		**LFPI**		**Sham**		**LFPI**		**Sham**		**LFPI**	
		Mean +/- SEM	n	Mean +/- SEM	n	Mean +/- SEM	n	Mean +/- SEM	n	Mean +/- SEM	n	Mean +/- SEM	n
		802.8 +/- 134.1	5	756 +/- 67.59	5	243 +/- 60.57	5	225 +/- 29.58	5	559.8 +/- 105.3	5	531 +/- 55.19	5
		t-test (p-value)				Mann-Whitney (p-value)				t-test (p-value)			
		0.7633				0.8016				0.8147			
	oGCL	**Fig. S4G**				**Fig. S4H**				**Fig. S4I**			
		**Sham**		**LFPI**		**Sham**		**LFPI**		**Sham**		**LFPI**	
		Mean +/- SEM	n	Mean +/- SEM	n	Mean +/- SEM	n	Mean +/- SEM	n	Mean +/- SEM	n	Mean +/- SEM	n
		208.8 +/- 62.96	5	163.8 +/- 9.178	5	118.8 +/- 38.54	5	66.6 +/- 5.4	5	90 +/- 32.57	5	97.2 +/- 7.742	5
		t-test (p-value)				Mann-Whitney (p-value)				t-test (p-value)			
		0.4995				0.1667				0.8351			
	Mol	**Fig. S4M**				**Fig. S4N**				**Fig. S4O**			
		**Sham**		**LFPI**		**Sham**		**LFPI**		**Sham**		**LFPI**	
		Mean +/- SEM	n	Mean +/- SEM	n	Mean +/- SEM	n	Mean +/- SEM	n	Mean +/- SEM	n	Mean +/- SEM	n
		1251 +/- 237.2	5	1345 +/- 151.8	5	570.6 +/- 71.13	5	561.6 +/- 101.7	5	680.4 +/- 177.8	5	783 +/- 102.4	5
		t-test (p-value)				t-test (p-value)				t-test (p-value)			
		0.7482				0.944				0.6306			
DCX	SGZ + GCL	**Fig. S3D**				**Fig. S3E**				**Fig. S3F**			
		**Sham**		**LFPI**		**Sham**		**LFPI**		**Sham**		**LFPI**	
		Mean +/- SEM	n	Mean +/- SEM	n	Mean +/- SEM	n	Mean +/- SEM	n	Mean +/- SEM	n	Mean +/- SEM	n
		t-test (p-value)				t-test (p-value)				t-test (p-value)			
BrdU	SGZ	**Fig. S3G**				**Fig. S3H**				**Fig. S3I**			
		**Sham**		**LFPI**		**Sham**		**LFPI**		**Sham**		**LFPI**	
		Mean +/- SEM	n	Mean +/- SEM	n	Mean +/- SEM	n	Mean +/- SEM	n	Mean +/- SEM	n	Mean +/- SEM	n
		5085 +/- 636.7	5	5575 +/- 284.3	5	2626 +/- 237	5	2938 +/- 240.8	5	2459 +/- 2637	5	2637 +/- 100.7	5
		t-test (p-value)				t-test (p-value)				t-test (p-value)			
		0.5025				0.3836				0.7447			
	Hilus	**Fig. S4D**				**Fig. S4E**				**Fig. S4F**			
		**Sham**		**LFPI**		**Sham**		**LFPI**		**Sham**		**LFPI**	
		Mean +/- SEM	n	Mean +/- SEM	n	Mean +/- SEM	n	Mean +/- SEM	n	Mean +/- SEM	n	Mean +/- SEM	n
		455.4 +/- 60.88	5	374.4 +/- 46.99	5	192.6 +/- 43.68	5	158.4 +/- 30.73	5	262.8 +/- 47.91	5	216 +/- 56.06	5
		t-test (p-value)				Mann-Whitney (p-value)				t-test (p-value)			
		0.323				0.6905				0.5434			
	oGCL	**Fig. S4J**				**Fig. S4K**				**Fig. S4L**			
		**Sham**		**LFPI**		**Sham**		**LFPI**		**Sham**		**LFPI**	
		Mean +/- SEM	n	Mean +/- SEM	n	Mean +/- SEM	n	Mean +/- SEM	n	Mean +/- SEM	n	Mean +/- SEM	n
		63 +/- 7.53	5	66.6 +/- 28.83	5	27 +/- 9	5	16.2 +/- 7.742	5	36 +/- 9.859	5	50.4 +/- 24.08	5
		t-test (p-value)				t-test (p-value)				t-test (p-value)			
		0.9068				0.3896				0.5951			
	Mol	**Fig. S4P**				**Fig. S4Q**				**Fig. S4R**			
		**Sham**		**LFPI**		**Sham**		**LFPI**		**Sham**		**LFPI**	
		Mean +/- SEM	n	Mean +/- SEM	n	Mean +/- SEM	n	Mean +/- SEM	n	Mean +/- SEM	n	Mean +/- SEM	n
		1013 +/- 163.3	5	984.6 +/- 152.3	5	406.8 +/- 81.02	5	543.6 +/- 142.8	5	606.6 +/- 116.5	5	441 +/- 41.44	5
		t-test (p-value)				t-test (p-value)				t-test (p-value)			
		0.9006				0.429				0.2174			

31 DPI - CONTRALATERAL

Marker	Region	Totals				Anterior				Posterior			
Ki67	SGZ	**Fig. S5A**				**Fig. S5B**				**Fig. S5C**			
		**Sham**		**LFPI**		**Sham**		**LFPI**		**Sham**		**LFPI**	
		Mean +/- SEM	n	Mean +/- SEM	n	Mean +/- SEM	n	Mean +/- SEM	n	Mean +/- SEM	n	Mean +/- SEM	n
		6795 +/- 198.8	5	7463 +/- 332.3	5	2959 +/- 319.6	5	3040 +/- 206.5	5	3836 +/- 188.2	5	4423 +/- 219.9	5
		t-test (p-value)				t-test (p-value)				t-test (p-value)			
		0.1229				0.8368				0.0772			
Ki67	Hilus	**Fig. S6A**				**Fig. S6B**				**Fig. S6C**			
		**Sham**		**LFPI**		**Sham**		**LFPI**		**Sham**		**LFPI**	
		Mean +/- SEM	n	Mean +/- SEM	n	Mean +/- SEM	n	Mean +/- SEM	n	Mean +/- SEM	n	Mean +/- SEM	n
		475.2 +/- 46.97	5	541.8 +/- 29.08	5	144 +/- 36.34	5	225 +/- 26.24	5	331.2 +/- 17.17	5	316.8 +/- 35.82	5
		t-test (p-value)				t-test (p-value)				t-test (p-value)			
		0.2624				0.1083				0.7264			
Ki67	oGCL	**Fig. S6G**				**Fig. S6H**				**Fig. S6I**			
		**Sham**		**LFPI**		**Sham**		**LFPI**		**Sham**		**LFPI**	
		Mean +/- SEM	n	Mean +/- SEM	n	Mean +/- SEM	n	Mean +/- SEM	n	Mean +/- SEM	n	Mean +/- SEM	n
		70.2 +/- 8.249	5	113.4 +/- 26.33	5	16.2 +/- 8.726	5	43.2 +/- 15.69	5	54 +/- 14.23	5	70.2 +/- 20.6	5
		t-test (p-value)				t-test (p-value)				t-test (p-value)			
		0.1561				0.1711				0.5358			
Ki67	Mol	**Fig. S6M**				**Fig. S6N**				**Fig. S6O**			
		**Sham**		**LFPI**		**Sham**		**LFPI**		**Sham**		**LFPI**	
		Mean +/- SEM	n	Mean +/- SEM	n	Mean +/- SEM	n	Mean +/- SEM	n	Mean +/- SEM	n	Mean +/- SEM	n
		1235 +/- 82.36	5	995.4 +/- 108.1	5	513 +/- 43.72	5	358.2 +/- 24.05	5	721.8 +/- 66.59	5	637.2 +/- 393.5	5
		t-test (p-value)				t-test (p-value)				t-test (p-value)			
		0.1162				0.0146				0.4647			
DCX	SGZ + GCL	**Fig. S5D**				**Fig. S5E**				**Fig. S5F**			
		**Sham**		**LFPI**		**Sham**		**LFPI**		**Sham**		**LFPI**	
		Mean +/- SEM	n	Mean +/- SEM	n	Mean +/- SEM	n	Mean +/- SEM	n	Mean +/- SEM	n	Mean +/- SEM	n
		39374 +/- 1381	5	38768 +/- 1763	5	18396 +/- 1063	5	20230 +/- 732.3	5	20878 +/- 2418	5	18581 +/- 1297	5
		t-test (p-value)				t-test (p-value)				t-test (p-value)			
		0.7934				0.1931				0.4268			
BrdU	SGZ	**Fig. S5G**				**Fig. S5H**				**Fig. S5I**			
		**Sham**		**LFPI**		**Sham**		**LFPI**		**Sham**		**LFPI**	
		Mean +/- SEM	n	Mean +/- SEM	n	Mean +/- SEM	n	Mean +/- SEM	n	Mean +/- SEM	n	Mean +/- SEM	n
		1091 +/- 44.96	10	1048 +/- 67.9	10	700.2 +/- 38.55	10	666 +/- 59.38	10	386.1 +/- 30.31	10	381.6 +/- 27.86	10
		t-test (p-value)				Mann-Whitney (p-value)				t-test (p-value)			
		0.6022				0.8108				0.9142			
BrdU	Hilus	**Fig. S6D**				**Fig. S6E**				**Fig. S6F**			
		**Sham**		**LFPI**		**Sham**		**LFPI**		**Sham**		**LFPI**	
		Mean +/- SEM	n	Mean +/- SEM	n	Mean +/- SEM	n	Mean +/- SEM	n	Mean +/- SEM	n	Mean +/- SEM	n
		115.2 +/- 18.39	10	141.3 +/- 28.27	10	50.4 +/- 13.16	10	51.3 +/- 12.87	10	63 +/- 8.161	10	90 +/- 21.17	10
		Mann-Whitney (p-value)				t-test (p-value)				t-test (p-value)			
		0.7516				0.9615				0.2495			
BrdU	oGCL	**Fig. S6J**				**Fig. S6K**				**Fig. S6L**			
		**Sham**		**LFPI**		**Sham**		**LFPI**		**Sham**		**LFPI**	
		Mean +/- SEM	n	Mean +/- SEM	n	Mean +/- SEM	n	Mean +/- SEM	n	Mean +/- SEM	n	Mean +/- SEM	n
		75.6 +/- 12.74	10	54 +/- 10.82	10	36 +/- 7.707	10	30.6 +/- 8.818	10	39.6 +/- 9.506	10	23.4 +/- 4.49	10
		t-test (p-value)				t-test (p-value)				t-test (p-value)			
		0.2126				0.6503				0.1407			
BrdU	Mol	**Fig. S6P**				**Fig. S6Q**				**Fig. S6R**			
		**Sham**		**LFPI**		**Sham**		**LFPI**		**Sham**		**LFPI**	
		Mean +/- SEM	n	Mean +/- SEM	n	Mean +/- SEM	n	Mean +/- SEM	n	Mean +/- SEM	n	Mean +/- SEM	n
		301.5 +/- 58.71	10	257.4 +/- 21.14	10	154.8 +/- 48.61	10	122.4 +/- 21.56	10	139.5 +/- 17.56	10	135 +/- 15.93	10
		Mann-Whitney (p-value)				Mann-Whitney (p-value)				Mann-Whitney (p-value)			
		0.7246				0.9558				0.6669			
